# Draft Genome Sequences of Two *Vibrio splendidus* Strains, Isolated from Seagrass Sediment

**DOI:** 10.1128/genomeA.01769-15

**Published:** 2016-02-18

**Authors:** Ruth D. Lee, Guillaume Jospin, Jenna M. Lang, Jonathan A. Eisen, David A. Coil

**Affiliations:** a University of California Davis Genome Center, Davis, California, USA; b University of California Davis, Department of Evolution and Ecology, Department of Medical Microbiology and Immunology, Davis, California, USA

## Abstract

Here, we present the draft genome sequences of *Vibrio splendidus* UCD-SED7 and UCD-SED10 (phylum *Proteobacteria*). These strains were isolated from sediment surrounding *Zostera marina* roots near the UC Davis Bodega Marine Laboratory (Bodega, Bay, California). These assemblies contain 5,334,236 bp and 5,904,824 bp, respectively.

## GENOME ANNOUNCEMENT

Both *Vibrio splendidus* UCD-SED7 and UCD-SED10 strains were isolated from sediment surrounding common eelgrass (*Zostera marina*) roots near the UC Davis Bodega Marine Laboratory (Bodega Bay, CA, USA). The sampling site was located north of Westshore Park, California (38°19′10.0″N, 123°03′13.8″W).

*V. splendidus* is a common Gram-negative marine bacterium, prevalent enough in coastal habitats to be cultured from open waters (1, 2). Previous studies have also observed bioluminescence in this species; its luminescence is visible to the naked eye in *V. splendidus* bacterial aggregates and in *V. splendidus*-associated plankton (2).

Dilutions (1:100 and 1:1,000) of sediment were made and spread on a modified seawater nutrient agar medium (ATCC Medium 2205, using InstantOcean in place of synthetic seawater), grown at room temperature for 24 h, and individual colonies were double dilution streaked. A Wizard genomic DNA purification kit (Promega) was used to extract DNA from fresh 5-mL seawater nutrient media overnight cultures.

A Nextera DNA sample prep kit (Illumina) was used to make paired-end libraries (Illumina). Libraries were sequenced on an Illumina MiSeq, at a read length of 300 bp. A total of 2,717,081 and 910,320 high-quality paired-end reads were processed by the A5-miseq assembly pipeline for strains UCD-SED7 and UCD-SED10, respectively (3, 4). This pipeline automates error correction, contig assembly, data cleaning, quality control, and scaffolding. The resulting assemblies consisted of 61 contigs for UCD-SED7 (longest: 1,279,008 bp; *N*_50_: 589,986) and 162 contigs for UCD-SED10 (longest: 364,583; *N*_50_: 105,476) that were submitted to GenBank. The final assembly of UCD-SED7 contained 5,334,236 bp with a G+C content of 44.1% and an overall coverage estimate of ~255×. The final assembly of UCD-SED10 contained 5,904,824 bp with a G+C content of 44.1% and an overall coverage estimate of ~77×. Genome completeness was assessed using the PhyloSift software (5), which searches for a list of 37 highly conserved, single-copy marker genes (6), of which all 37 were found in both assemblies.

The RAST server was used to perform automated annotations on both strains (7–9). *V. splendidus* UCD-SED7 contains 4,595 predicted protein-coding sequences and 182 predicted noncoding RNAs. *V. splendidus* UCD-SED10 contains 5,220 predicted protein-coding sequences and 162 predicted noncoding RNAs.

The 16S rRNA sequences were obtained from the RAST annotation and used to attempt to identify the strains via BLAST and phylogenetic trees (10). However, each of these assemblies contained three distinct 16S rRNA sequences, suggesting significant copy variation. Because these heterogeneous copies have different placements into a phylogenetic tree, we instead built a whole-genome tree of all available *Vibrio* genomes. This tree, to be described elsewhere (our unpublished data) gave an unambiguous placement of UCD-SED7 and UCD-SED10 within a monophyletic clade of *V. splendidus*.

### Nucleotide sequence accession numbers.

Both whole-genome shotgun projects have been deposited at DDBJ/EMBL/GenBank under the accession numbers LIZK00000000 (for UCD-SED7) and LIZL00000000 (for UCD-SED10). The versions described in this paper are versions LIZK00000000.1 (for UCD-SED7) and LIZL00000000.1 (for UCD-SED10).
